# Engineering CAR-NK cells: how to tune innate killer cells for cancer immunotherapy

**DOI:** 10.1093/immadv/ltac003

**Published:** 2022-02-03

**Authors:** Dayane Schmidt, Sima Ebrahimabadi, Kauan Ribeiro de Sena Gomes, Graziela de Moura Aguiar, Mariane Cariati Tirapelle, Renata Nacasaki Silvestre, Júlia Teixeira Cottas de Azevedo, Dimas Tadeu Covas, Virginia Picanço-Castro

**Affiliations:** 1 Regional Blood Center of the School of Medicine of Ribeirão Preto, University of São Paulo, Ribeirão Preto, Brazil; 2 Department of Medical Imaging, Hematology, and Clinical Oncology, Ribeirão Preto Medical School, University of São Paulo, Ribeirão Preto, São Paulo, Brazil

**Keywords:** NK cells, chimeric antigen receptor, cell therapy, genetic engineering, cancer

## Abstract

Cell therapy is an innovative approach that permits numerous possibilities in the field of cancer treatment. CAR-T cells have been successfully used in patients with hematologic relapsed/refractory. However, the need for autologous sources for T cells is still a major drawback. CAR-NK cells have emerged as a promising resource using allogeneic cells that could be established as an off-the-shelf treatment. NK cells can be obtained from various sources, such as peripheral blood (PB), bone marrow, umbilical cord blood (CB), and induced pluripotent stem cells (iPSC), as well as cell lines. Genetic engineering of NK cells to express different CAR constructs for hematological cancers and solid tumors has shown promising preclinical results and they are currently being explored in multiple clinical trials. Several strategies have been employed to improve CAR-NK-cell expansion and cytotoxicity efficiency. In this article, we review the latest achievements and progress made in the field of CAR-NK-cell therapy.

## Introduction

Cell therapy using T-cells engineered to express a chimeric antigen receptor (CAR-T cells) has resulted in outstanding improvements in the treatment of patients with hematological cancer [1–3]. CAR structure comprises an extracellular domain, hinge, transmembrane region, and intracellular signaling domains. The use of a single-chain variable fragment (scFv) as the recognition domain directs T-cells to specific tumor antigens, without the need for HLA presentation allowing a broader application [4]. Despite its potential, the wide use of CAR-T-cell therapy presents many challenges in cancer treatment. It can lead to severe side effects, such as cytokine release syndrome (CRS) and neurotoxicity [5, 6]. Moreover, its extensive application is limited by inherent risks such as graft versus host disease (GvHD) and the quality of patient T cells that are used to produce CAR-T cells [7]. A promising alternative is allogeneic cell therapy using NK cells expressing CAR (CAR-NK cells).

NK cells are innate immunity cytotoxic lymphocytes that can eliminate virus-infected and tumor cells [8]. They also secrete cytokines which can activate other immune cells [9]. One of the advantages of using these cells in CAR**-**based therapies is that they preserve their normal function against cancer cells, which might prevent tumor evasion by downregulation of CAR target [10]. Also, NK cells present a short half-life in circulation, increasing their safety [11]. To exert their function, they are independent of HLA presentation which decreases the risks of GvHD [12]. This allows for the use of NK cells from allogeneic sources and might enable the establishment of CAR-NK cells as an off-the-shelf therapy. Despite the possible benefits with CAR-NK cells, there are still many challenges to attain a robust clinical application.

Some of the issues for CAR-NK cells development are the inefficient *in vitro* expansion, low efficiency of genetic modification, and that most studies use CAR constructs designed for CAR-T cells.

In addition, due to the NK cells’ relatively short half-life, it may be necessary to administer more than one dose of CAR-NK cells in patients, but long-term follow-up studies with patients treated with CAR-NK still need to be conducted. Lastly, it is still a challenge to overcome the solid tumor microenvironment (TME) and the development of CAR-NK cells that can migrate properly to the tumor and to escape from the immunosuppressive effect of TME is extremely necessary [13]. In this review, we explore improvements in CAR-NK-cell therapy, including genetic engineering strategies to develop NK-cell-specific CAR.

## NK sources and expansion

The success of CAR-NK-cell therapy depends on multiple factors, such as the choice of the NK source, the expansion method, the vector used to deliver and express the CAR, and the design of the CAR molecule. Table 1 lists the advantages and disadvantages of the main factors that can affect CAR-NK-cells production that will be discussed in this review.

**Table 1.** Advantages and disadvantages of new technologies for CAR-NK production

	Advantages	Disadvantages
NK cell type		
NK-92	• Easy to expand and to engineer; • Homogenous product; • Low *in vivo* persistence.	• Safety risk; • Requires irradiation; • No CD16 expression.
Peripheral Blood	• Easy to obtain; • Does not require irradiation; • Highly cytotoxic.	• Difficult expansion; • Low transduction; efficiency; • Sensitive to freeze/thaw cycles; • Non homogenous.
Cord blood	• Ease of collection; • Fewer T cells; • Presence of unique NK progenitors; • High proliferation capacity.	• Difficult expansion; • Non homogenous product; • Immature cells.
iPSC	• Yields more cells; • Easy to engineer; • Homogenous product.	• Longer production period; • Immature phenotype; • Low *in vivo* persistence; • Potentially immunogenic; • Potentially tumorigenic.
NK expansion methods		
Cytokines combination	• Promotes differentiation of memory-like natural killer cells.	• Requires high initial number of cells; • High cost; • Increases the chance of Treg activation.
Synthetic beads/antibodies	• Easy handling; • Easy to scale up.	• Low to moderate expansion; • Expensive.
Feeder cells	• Efficient activation and high expansion.	• Complex co-culture system.
Membrane particles	• High expansion rates.	• Laborious process of fabrication and characterization; • Risk of residual stimulatory cell material in the final product
Gene delivery		
Retroviruses	• Permanent modification of cells.	• Requires actively dividing cells; • Random integration profile (Risk of insertional mutagenesis); • Potential of replication competent retrovirus (RCR); • High manufacturing cost of GMP-grade vectors.
Lentiviruses	• Transduction of non-dividing cells; • Permanent modification of cells.	• Random integration profile (risk of insertional mutagenesis); • Potential of replication competent retrovirus (RCL); • High manufacturing cost of GMP-grade vectors.
Transposons	• Cost-effective; • Easier to produce on a large scale; • Large insert capacity; • Stable transgene expression.	• High cell death rates; • Low integration rate; • Risk of insertional mutagenesis.
CRISPR/Cas 9 technology	• Site-specific integration of gene of interest; • Permanent expression of CAR	• Possible off-target effects; • Low delivery efficiency; • Licensing restrictions
mRNA	• Low risk of insertional mutagenesis; • High efficiency of genetic material delivery.	• Inherently labile; • Short period of expression.
Episomes	• Stable expression; • Cost-effective; • Low risk of insertional mutagenesis; • Safety profile compared to viral methods.	• A good delivery method is still needed.

Currently, NK cells used in cancer immunotherapy can be manufactured from diverse sources, such as cell lines, peripheral blood (PB), bone marrow, umbilical cord blood (CB), and induced pluripotent stem cells (iPSC) [14–18].

NK cell lines have been used in cancer immunotherapy because of their increased expansion ability *in vitro* and their relatively simple cultivation conditions. To date, there are seven human NK cell lines: HANK-1, KHYG-1, NK-92, NK-YS, NKL, SNK-6, and YT. Immunophenotypic characterization has revealed the immaturity of the cell lines tested, defined as CD16^−^CD56^+^. Only KHYG-1 and NK-92 show significant activity against the MHC-I negative target cell line K562. This discrepancy may be because other NK cell lines have not yet reached the maturity stage where they acquire typical NK cytotoxicity [19]. The only FDA-approved cell line for clinical trials is the NK-92, which is highly cytotoxic [20].

Most studies with CAR-NK cells have used enriched cells from PB from allogeneic donors as their source [21]. In PB, NK cells represent a low percentage of circulating lymphocytes. NK cells are defined as two functionally distinct subsets: CD56^bright^ CD16^−^ and CD56^dim^CD16^+^ cells. In PB, the ratio between CD56^bright^ and CD56^dim^ is about 1:9 respectively. The major differences between these two subsets are that CD56^dim^ NK cells show significantly higher cytotoxic activity and contain much more perforin and granzyme while CD56^bright^ are more efficient producers of pro-inflammatory cytokines [22–24]. Also, the pattern of surface receptor expression differs between the CD56^bright^ and CD56^dim^ populations. CD56^dim^ express CD16 and inhibitory KIR while CD56^bright^ are negative for CD16 and KIR but positive for NKG2A and the IL-2 receptor α chain (IL-2Rα/CD25) [25,26].

An alternative source of NK cells to PB is CB. Clinical numbers of CB-derived NK cells (2 × 10^9^) with high purity (92% CD56^+^) were reached using a bioreactor in a GMP**-**compliant system [27]. Some benefits of using CB-NK-cells are the relative ease of collection and the reduced risk of GvHD since CB contains reduced T-cell counts [28,29].

Different cytokines have been used to improve NK-cell growth in cell culture, such as IL-2, IL-12, IL-15, IL-18, and IL-27, which are related to cell proliferation, as well as cytotoxic potential [30,31].

NK-cells isolated from PB have been expanded using IL-2 and IL-15, with and without the addition of IL-21, which generated different NK subpopulations. CD56^dim^ population was higher in the presence of IL-21, while NKG2D and NKp44 cell surface markers were downregulated [32]. The combination of IL-12, IL-15, and IL-18 to expand PB**-**NK cells results in memory-like NK cells with high cytotoxicity and increased production of interferon-γ [33]. Anti-CD19 CAR-NK expanded with these cytokines showed enhanced anti-tumor activity in a target**-**specific manner [34]. Liu et al. [21]have recently investigated 16 different combinations of the cytokines IL-2, IL-12, IL-15, IL-18, and IL-21 for PB**-**NK-cell expansion, and the combination of IL-2, IL-15, and IL-18 resulted in improved expansion and cytotoxicity [31]. Another study evaluating PB**-**NK-cell expansion demonstrated that the cytokines IL-15, IL-18, and IL-27 were the optimal combination leading to enhanced cytotoxicity [30].

CB cells are an excellent source to generate off-the-shelf products, due to their less restrictive requirements for HLA matching and lower risks to cause GvHD [35]. Cord blood is an even better source of NK cells than PB, with 18.2% of lymphocytes being NK cells, while we found 12.7% of NKs in PB [36]. NK cells from CB can be successfully transduced with CAR specific for CD19 and are potentially more cytotoxic to tumor cells from patients with Chronic Lymphocytic Leukemia (CLL) than non-transduced NK cells [37].

Spanholtz et al. [28] described a cytokine**-**based culture system for *ex vivo* expansion of NK-cells from hematopoietic stem cells from CB. CD56^+^CD3^−^ cells were generated from CD34^+^ CB-cells. The generated NK-cells expressed NKG2A and KIR receptors, and high levels of NKG2D activating receptors. Functional analysis showed NK-cells are cytotoxic against myeloid leukemia cell lines, melanoma cell lines, and primary Acute Myeloid Leukemia (AML) cells [38].

However, although many cytokines have been implemented to improve NK expansion, cell growth is still very modest [39]. Another approach that has been used for NK-cell expansion is adding feeder cells to its culture, such as K562-mb15-41BBL cells [40]. The K562-mb15-41BBL feeder cell was made by transducing K562-cells with constructs encoding the ‘membrane-bound’ form of IL-15 (mbIL15) and human 4-1BB ligand (4-1BBL) [40,41], which improves NK-cell growth and cytotoxic capacity. NK-cells co-cultivated with K562-mb15-41BBL promoted a mean NK expansion of 277-fold in 21 days [42]. Alternatively, NK cells were co-cultured with K562-based artificial APC expressing membrane**-**bound IL-21 (mbIL21) or mbIL15, showing a higher fold expansion with mbIL21 after 3 weeks. As well as this, NK-cell expansion with mbIL21 resulted in an increase in telomere length compared to mbIL15, indicating it can diminish NK-cell senescence [43]. K562 engineered to express CD48, 4-1BBL, and mbIL21 has been also used as a potent feeder cell, allowing clinical scale production of NK cells [44]. CD48 is the counter receptor for 2B4, which participates in a variety of cell-to-cell interactions and is an important activator of NK cells [45], while 4-1BBL, the counter-receptor for CD137, mediates NK-cell proliferation and differentiation [46,47].

Other cell types have been tested as feeders for primary NK-cell expansion, and include OCI-AML-3 cells overexpressing mbIL21 (known as NKF cells). NKF-cells showed higher fold expansion at a 5:1 (NKF to NK) ratio and the NK expanded with NKF showed potent cytotoxicity comparable to NK-cells co-cultured with mbIL21-K562 [48].

NK-92 engineered to express OX40L and to secrete neoleukin-2/15 (Neo-2/15) has also been used for expansion of PB**-**NK cells leading to a 2180-fold expansion in 21 days [49]. Neo-2/15 is a newly engineered protein that mimics the function of both IL-2 and IL-15 [50]. Lastly, NK-cells from CB co-cultivated with irradiated Epstein-Barr virus-transformed lymphoblastoid cell line (EBV-LCL) showed high expansion levels and demonstrated to also be a feasible approach [51].

Although feeder cell-based NK-cell expansion systems can be used to obtain good products [52–54], the use of a feeder cell line may result in unpredictable risks [55]. An alternative method to feeder cells is the use of plasma membrane-derived particles from K562-mbIL15-41BBL, which induced a 250-fold expansion of highly cytotoxic NK-cells after 17 days [56]. An alternative method to promote NK-cell expansion without feeder cells is monoclonal antibodies (mAb), such as the combination of anti-CD52 and anti-CD3, which demonstrated a favorable growth of NK-cells while suppressing CD4^+^T-cells from peripheral blood mononuclear cell (PBMC) [57]. Moreover, activation using microbeads covered with antibodies against various NK receptors, such as NKp46, 2B4, DNAM-1, CD2, and CD18, is another strategy that can improve NK-cell expansion and activation [58].

## Delivery methods for engineering NK-cells

### Viral vectors

Lentiviruses and retroviruses are the most used systems to induce stable expression of CAR in NK cells. However, the genetic manipulation of NK cells has historically been limited by the induction of apoptosis in NK cells after genetic manipulation and the low efficiency of transgene delivery compared to T cells [59].

In order to facilitate viral transduction, some approaches have been exploited, including (i) changing the electrical charges of cells, (ii) increasing interaction between virus and target cell via integrin binding, (iii) up**-**regulation of low-density lipoprotein receptors (LDLR), (iv) changing the viral envelope, and (v) inhibition of innate immune signaling.

Both viral envelope glycoproteins and target cell receptors may contain negative charges that can be detrimental to transduction [60]. Polycationic reagents such as lipids, polymers, and peptides can induce the aggregation of viral particles and facilitate binding to cells through modulation of electrostatic interactions, an example is a polybrene [61]. Retronectin is a chimeric peptide that binds to integrins VLA-4 and VLA-5 and also to the virus. Its use results in an increased transduction efficiency of NK cells with different retroviral platforms. Transduction using alpha-retroviral particles in combination with retronectin allowed stable transduction with an increase of 90% in NKL cell line and up to 60% in primary NK cells [62].

The most used viral envelope is the one pseudotyped with the Vesicular Stomatitis Virus type-G (VSV-G) envelope glycoprotein. The main receptors for this envelope protein are LDLR and phosphatidylserine [63]. However, NK-cell lines and primary NK-cells express low levels of LDLR, and up**-**regulation of LDLR expression in NK cells by lipophilic drugs, like rosuvastatin, leads to increased transduction rate [64].

In addition, replacing VSV-G with Baboon envelope pseudotyped lentiviral vectors (BaEV-LV) increased the transduction rate of freshly isolated human NK-cells [65].

NK cells are responders to viral infections [66] and this can be reflected in the reduced viral transduction efficiency observed in NK cells. During viral transduction, intracellular antiviral defense mechanisms including one or more of the receptors RIG-I, MDA-5, and TLR3 transmit signals to TBK1 via multiple proteins, leading to the production of IFN-γ that can contribute significantly to the resistance of NK cells to lentiviral genetic modification. Receptors involved in antiviral responses are highly expressed in NK cells and the use of the TBK1/IKKɛ complex inhibitor BX795 improves the transduction rates in NK cells [67,68].

### Non-viral vectors

Although viral transduction remains the most employed gene delivery method in NK cells, several limitations still exist and the risk of vector integration into the genomic DNA is the most concerning. Non-viral vectors can be classified into integrative and non-integrative vectors.

Transposons, mRNA, and episomal vectors are examples of non-viral integrative systems. The transposon system, such as Sleeping Beauty (SB), piggyBac, or Tol2, are effective non-viral vectors for delivering genetic material. The transposase enzyme recognizes transposon-specific inverted terminal repeat sequences (ITR) located at both ends of a transposon vector and then simply cuts the sequence and binds it somewhere in the target DNA. This creates stable/permanent genomic integration into the host cell [69].

An SB transposon vector was successfully used to express anti-mesothelin CAR in NK cells derived from iPSC. The resulting CAR-NK-iPSC cells successfully mediate strong anti-tumor activity, repressed tumor growth, and prolonged survival [70]. Wang et al. [71] engineered CAR-NK-cells expressing a chimeric receptor with NKG2D ectodomain and DAP10-CD3ζ signaling domain by transfecting cells with a piggyBac vector complexed with PBAE polymer. With this system, cells maintained high viability after transfection. Combining these CAR-NK-cells with the blockade of CD73 displayed synergic efficacy against CD73^+^ lung cancer [71]. Transposons are easier to produce on a large scale and they harbor greater transgenic capacity. Their integration occurs generally in AT-rich genomic regions [72] and they are considered biologically safe. However, this year two patients  developed malignant lymphoma after treatment with CAR-T-cells genetically modified with a piggyBac vector [73].

New innovative methodologies have been developed to overcome the risk of mutagenesis through the insertion of integrative vectors. Other non-integrative gene delivery methodologies, such as mRNA and episomal vectors, have emerged as a new delivery form that can be used to deliver CAR to NK cells.

The electroporation of mRNA-encoding CARs into NK cells has been successfully demonstrated with an efficiency of up to 81% [74]. This method has also been used to engineer CAR-NK-cells targeting the CD20 antigen in B-cell non-Hodgkin’s lymphoma [75] and Burkitt’s lymphoma [76]. In addition, NK-cells transfected with anti-ROR1 CAR mRNA have been used for metastatic solid tumors treatment [77, 78]. This innovative approach is also being tested in clinical trials with CARs targeting CD19 and NKG2DL (NCT00995137 and NCT03415100) (Table 2).

**Table 2.** Clinical trials using CAR-NK cells for gene therapy

Reference	NK source	Gene transfer	Target molecule	Target tumor	CAR construct	Status	Sponsor	Study Phase
NCT04796675	CB	RV	CD19	CD19^+^ B-cell malignancies	Unknown	Recruiting	Wuhan Union Hospital, China	I
NCT03056339 [125]	CB	RV	CD19	B Lymphoid Malignancies	CD19-CD28-zeta-2A-iCasp9-IL15	Recruiting	M.D. Anderson Cancer Center	I/II
NCT03656705	NK92	RV/LV	Unknown	Non-small Cell Lung Cancer	Unknown	Enrolling by invitation	Xinxiang medical university	I
NCT02944162 [156]	NK92	LV	CD33	AML	ScFv-CD28-CD137-CD3z	Unknown	PersonGen BioTherapeutics (Suzhou) Co., Ltd.	I/II
NCT02839954	NK92	LV	MUCI	Solid tumor	ScFv-CD28-CD137-CD3z	Unknown	PersonGen BioTherapeutics (Suzhou) Co., Ltd.	I/II
NCT02892695	NK92	LV	CD19	Lymphoma, leukemia	ScFv-CD28-CD137-CD3z	Unknown	PersonGen BioTherapeutics (Suzhou) Co., Ltd.	I/II
NCT03383978 [157]	NK92	LV	HER2	GBM	ScFv-CD28-CD3z	Recruiting	Johann Wolfgang Goethe University Hospital	I
NCT03940833	NK92	LV	BCMA	Multiple myeloma	Unknown	Recruiting	Asclepius Technology Company Group (Suzhou) Co., Ltd.	I/II
NCT03941457	NK92	LV	ROBO1	Pancreatic Cancer	Unknown	Recruiting	Asclepius Technology Company Group (Suzhou) Co., Ltd.	I/II
NCT03940820	NK92	LV	ROBO1	Solid Tumor	Unknown	Recruiting	Asclepius Technology Company Group (Suzhou) Co., Ltd.	I/II
NCT04245722 [147]	iPSC	LV	CD19	B-cell lymphoma, CLL	scFv-NKG2D-2B4-CD3z-IL-15/RhnCD16	Recruiting	Fate Therapeutics	I
NCT00995137	PB-NK	mRNA electroporation	CD19	B-ALL	ScFv-CD8aTM-CD137-CD3z	completed	St. Jude Children’s Research Hospital	I
NCT02742727	NK92	Electroporation	CD7	Lymphoma, leukaemia	ScFv-CD28-CD137-CD3z	Unknown	PersonGen BioTherapeutics (Suzhou) Co., Ltd.	I/II
NCT03415100 [115]	PB-NK	mRNA electroporation	NKG2DL	Metastatic solid tumor	ScFv-CD8aTM-CD3z; ScFvCD8aTM-DAP12	Unknown	The Third Affiliated Hospital of Guangzhou Medical University	I

Episomal vectors are emerging as a safer alternative to integrating vectors [79]. Plasmid vectors containing S/MAR (matrix support and fixation region) enable plasmid retention and replication within the host cell nucleus [80]. Recently, this type of vector was used to genetically modify T-cells with an anti-CD19 CAR resulting in CAR-T-cells with long-term transgene expression and *in vivo* cytotoxicity [81].

CRISPR-Cas9 is a technology that can also be used in non-viral vectors and unlike transposons, the insertion of genetic material takes place in a specific location. In NK cells, knock-in with CRISPR-Cas9 has been implemented by combining with adeno-associated virus (AAV) delivery of template DNA for homologous repair [82]. Kararoudi et al. used AAV delivery of template DNA with CRISPR-Cas9 to generate anti-CD33 CAR-NK cells, which demonstrated maintenance of CAR expression after 2 weeks in culture and efficient targeting of AML cells [83]. For non-viral DNA template delivery, the combination of truncated Cas9 target sequences (tCTS) added to the end of homology-directed repair (HDR) template with an anionic polymer can increase knock-in efficiency in NK cells from 3.09% to 16.6% [84], although this strategy has not been used for CAR delivery yet.

### Methods to delivery non-viral vectors

There are different methods to physically deliver naked genetic material, those methods can be combined with gene carriers or enhancement buffers, such as biphasic polymers, liposomes, or other vehicles [85–87]. These strategies create a temporary pore in the cell membrane or provide carriers that can fuse with the cell membrane allowing incorporation.

Electroporation-based methods are well known for transfecting mammalian cells with foreign genetic material. However, the possibility of permanent cell damage due to the electroporation process is still a challenge [88]. To minimize the damage caused by the electroporation, other methods to permeabilize the cell membrane have been developed and employed to produce CAR-NK cells. Cell squeezing is an intracellular delivery method based on rapid mechanical deformation of the cell’s membrane as the cell flows at high speed through a narrow microchannel in a silicon chip, the transient pores created during the ‘squeezing’ allow for molecules in the cell’s surroundings to diffuse into its cytosol [89]. The technique has been shown to deliver a large panel of small or uncharged molecules such as dextrans, proteins, RNPs, nanoparticles, and siRNA [90–92], and does not disrupt cell function [90]. Squeezing cells followed by an electrical field enhanced the delivery of nucleic acids. This technique could allow cell transduction with mRNAs for safer CAR delivery in the context of NK cells. Chang et al. have developed a technique of nanochannel-electroporation and when used to deliver a CAR/GFP-reporter plasmid in NK cells, it led to around 80% of GFP expression with the maintenance of cell viability [93].

Each of the delivery methods discussed has a set of advantages and disadvantages compared with each other (Table 1). But the choice of an efficient delivery method also depends on other factors such as NK-cell source and CAR construct [94]. Table 2 lists CAR-NK-cells used in the clinical trials registered on Clinicaltrials.gov that provide information on the delivery method. Among the studies whose type of delivery system is known, three studies were performed with retroviral vectors, eight studies with lentiviral vectors, and three studies by electroporation.

## Specific CARs molecules for NK cells

Initially, CAR-NK-cells were constructed with only CD3ζ (first**-**generation CAR) as a signaling domain, and these were shown to be efficient in eliminating target cells [95, 96]. As for CAR-T cells, CAR-NK with one or two additional co-stimulatory domains (second**-** and third**-**generation), such as CD28 and/or 4-1BB along with CD3ζ, have also been successfully applied in NK cells [40, 97, 98]. Recently, fourth**-** and fifth**-**generations CAR have been described, both are based on second**-**generation CAR. Fourth**-**generation secretes cytokines, while the fifth contains an intracellular domain of a cytokine receptor [99,100].

Optimization with more specific signaling domains for NK cells has been pursued to increase its cytotoxicity. NK cell activation results from simultaneous stimulation of NK cell-activating receptors, such as natural cytotoxicity receptors (NCR), NKG2D, 2B4 (CD244), and DNAM-1 (CD226). NCR NKp30 and NKp46 associate with CD3ζ and FcRγ, while NKp44 interacts with DNAX-activating protein 12 (DAP12). These adaptors mediate NK-cell activation via their immunoreceptor tyrosine-based activation motifs (ITAM) [101–103]. CD3ζ signaling domain is also co-associated with CD16 in NK cells [104]. NKG2D interacts with DAP10, which presents a phosphatidylinositol-3 kinase (PI3K) binding motif [105]. 2B4 and DNAM-1 are not associated with adaptors containing ITAM and act as co-receptors amplifying signals induced by NCR and NKG2D [106, 107].

First**-**generation constructs with DAP12 or FcεR1γ (high-affinity immunoglobulin epsilon receptor subunit gamma) as the CAR signaling domain instead of CD3ζ, demonstrated that these specific molecules were efficient in activating CAR-NK cells [108, 109]. Most studies evaluate constructions with additional signaling domains to achieve better signaling potency. The incorporation of 2B4 in CAR-NK cells against CD19 or G2D resulted in increased cytotoxicity compared to a CAR containing only CD3ζ. It was also observed that CAR-NK-cells containing only 2B4 failed to induce activation [110]. A study comparing 4-1BB-CD3ζ and 2B4-CD3ζ CAR-NK cells against CD5 demonstrated that even though both constructs were functional, 2B4-CD3ζ CAR-NK cells had enhanced cytotoxicity [111]. A third**-**generation CAR constructs with the NK-cell-activating molecules 2B4 and DNAM-1 displayed greater cytotoxicity against hepatocellular cancer cells expressing GPC3 compared to CAR with only CD3ζ or CD28-CD3ζ [112].

The most thorough investigation of CAR constructs for NK cells, assessed combinations of CD16, NKp44, NKp46, and NKG2D transmembrane regions, as well as costimulatory domains 2B4, DAP10, DAP12, and 4-1BB in several combinations with CD3ζ. Among the second and third**-**generation constructs evaluated, the one with NKG2D transmembrane domain and 2B4 as co**-**stimulatory signaling demonstrated higher cytotoxicity against target cells [70]. Conversely, a study comparing second**-**generation CAR with 4-1BB or 2B4 or a third**-**generation with both, demonstrated equal cytotoxicity among constructs in targeting GD2^+^ Ewing sarcoma cells [113].

**Figure 1.**
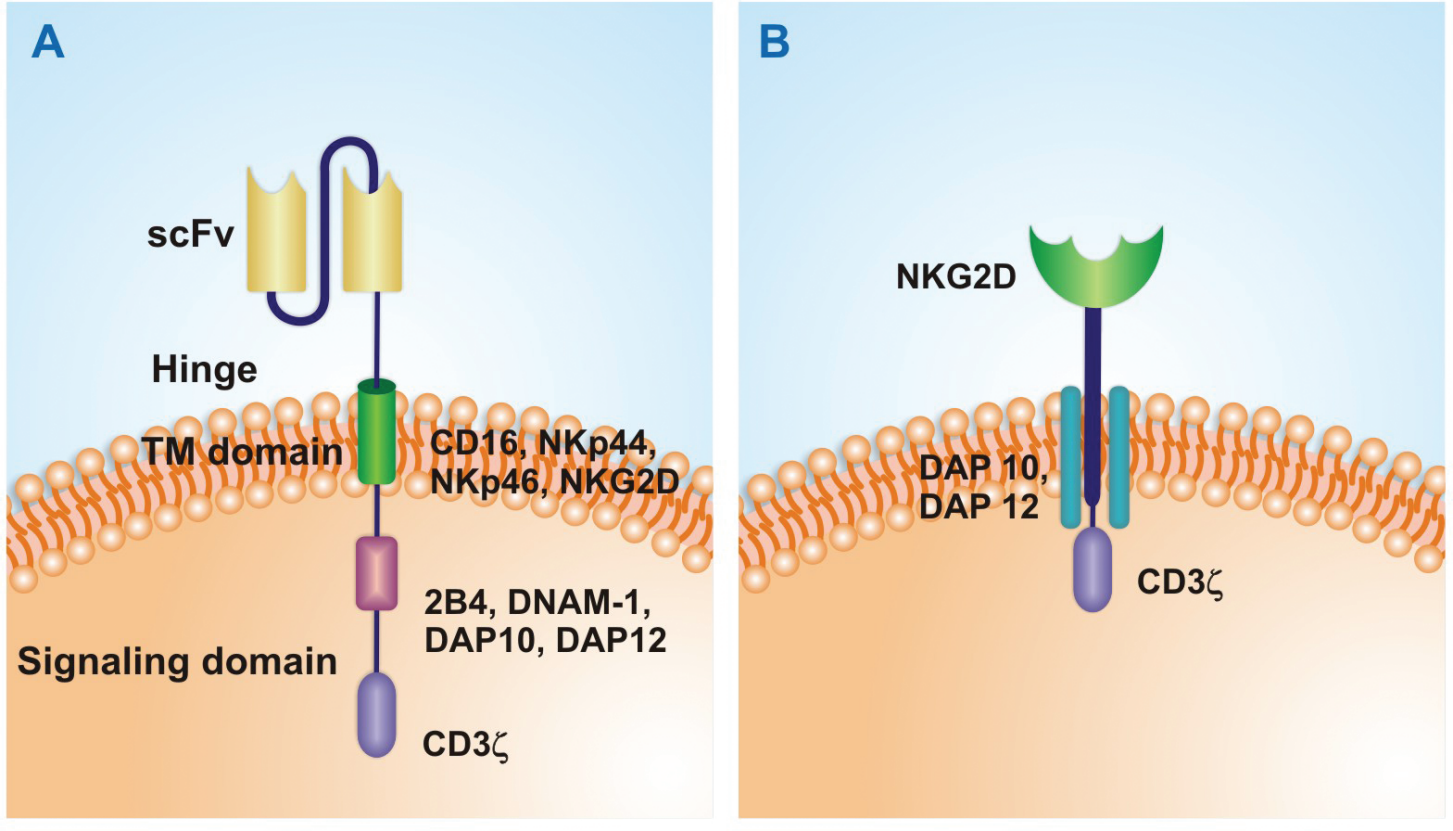
Specific CAR molecules for NK cells. (A) CAR with NK-specific signaling molecules. A second**-**generation CAR is represented, constructs specific for NK cells may contain 2B4, DNAM-1, DAP10, and/or DAP12 as co-stimulatory signaling domains with CD3ζ. The TM domain can also contain specific NK molecules such as CD16, NKp44, NKp46, or NKG2D. (B) NKG2D ectodomain as the recognition domain. A chimeric receptor containing NKG2D with DAP10/DAP12 and CD3ζ is represented. Constructions may contain the entire NKG2D protein as shown or only its ectodomain with a CD8 hinge and transmembrane region.

In addition to intracellular domain constructions, a chimeric receptor using NKG2D instead of a specific scFv has also been investigated. A NKG2D-DAP10-CD3ζ construct was able to increase NK-cells cytotoxicity against tumor cells without increasing activity against normal cells [114]. Chimeric receptors in NK cells using NKG2D ectodomain combined with DAP12 or CD3ζ signaling domains were both functional, but NK cells with the DAP12 construct had the highest cytolytic activity and were shown to be effective in patients with colorectal cancer [115]. Whereas a study comparing chimeric receptors for NK cells with NKG2D ectodomain combined with CD3ζ, CD28-CD3ζ or 4-1BB-CD28-CD3ζ showed higher cytotoxicity against ovarian cancer with the construct containing only CD3ζ [116]. Together these comparative studies indicate that CAR constructs including NK-specific molecules present a promising path in the development of CAR-NK-cell therapy (Fig. 1).

## Genetic engineering strategies beyond CAR target

### NK-cells engineered to express a non-cleavable CD16 Fc receptor

In the presence of IgG antibodies, NK-cells recognize target cells through the interaction between CD16A receptor and IgG, resulting in antibody-dependent cell-mediated cytotoxicity (ADCC) [117, 118].

The metalloproteinase ADAM17 can cleave CD16A after NK cell activation by multiple stimuli [119]. This regulation can affect the ADCC efficiency response decreasing IFN-γ production by NK cells [120, 121].

**Figure 2.**
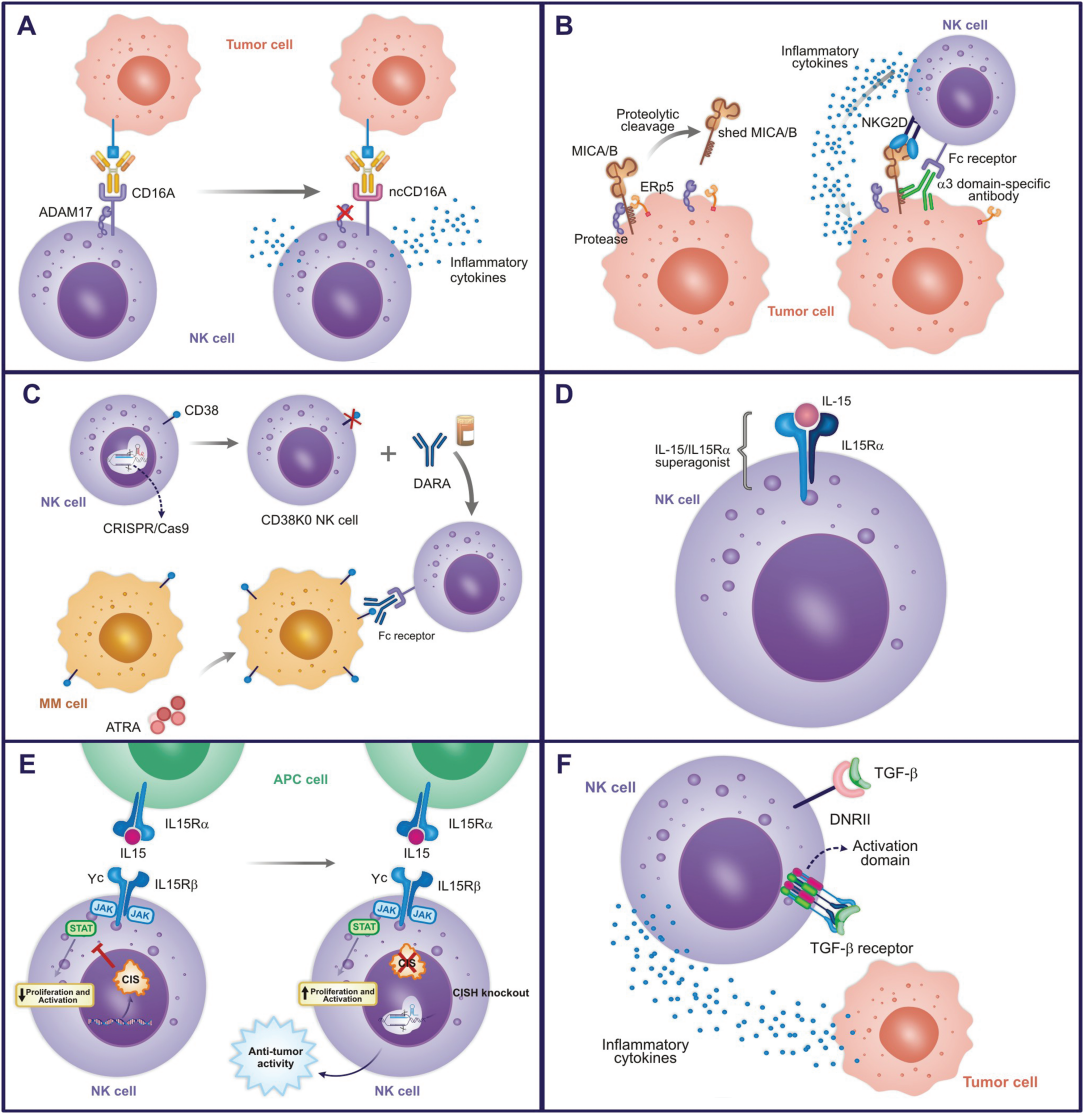
Genetic engineering strategies beyond CAR target. (A) NK-cells modified to express a non-cleavable CD16 Fc receptor through ADAM17 blocking. (B) Stabilization of MICA/B on the tumor cell surface using antibody against MICA α3 domain. Anti-MICA α3 domain further enhances NK function by NK cell Fc receptor recognition. (C) NK-cells CD38 KO associated with DARA for cancer treatment. CD38 is expressed in neoplastic B cells as well as in NK cells, monocytes, regulatory T cells, regulatory B cells, and myeloid-derived suppressor cells. Thus, knocking out CD38 in NK cells prevents NK cell depletion after treatment with DARA. (D) NK cells engineered to express IL-15 receptor fusion (IL-15/IL-15Rα) on cell surface. IL-15/IL-15-Rα complex is extremely important for generation, activation, and proliferation of NK cells. (E) NK CISH KO. The knock out of the *CISH* gene is another way of exploring the role of IL-15, it enhances sensitivity of NK cells to IL-15. (F) NK-cells expressing modified TGF-β receptor. TGF-β suppresses the function of NK cells and the use of the TGF-β receptor extracellular domain coupled to the intracellular domain of NK-cell-activating receptors has been associated with activation of these cells. In addition, TGF-β-DNRII expression in NK-cells resulted in the inhibition of TGF-β signaling.

An approach to block CD16 shedding involves the modification of the ADAM17 cleavage site in CD16A (between Val196 and Ser197), substituting Ser197 with Pro197 creating a cleavage resistance, referred to as non-cleavable CD16A (ncCD16A) (Fig. 2a) [122]. NK-cells expressing ncCD16A presented higher levels of ADCC and cytokine production in co-culture with various therapeutic mAb and tumor types, including ovarian cancer, Burkitt’s lymphoma, and lung adenocarcinoma [123]. Currently, a Phase I clinical trial is employing iPSC-NK cells expressing ncCD16A (FT516) in the treatment of acute myeloid leukemia (NCT04023071), as well as iPSC-NK cells expressing ncCD16A and anti-CD19CAR (FT596), as a combined targeting approach for the treatment of B-cell lymphoma (BCL) and CLL (NCT04245722) (Table 2) [123–125].

### NK-cells engineered to stabilize MICA/B on the tumor cell surface

NK-cells can recognize and eliminate tumor cells expressing MICA/B by the NKG2D receptor. MICA and MICB are stress-inducible ligands of NKG2D expressed on the cell surface. Their expression is strongly induced by cellular stress conditions due to DNA damage, viral infections, and neoplastic transformation, being absent in normal tissues [126, 127]. To escape NKG2D-mediated immune surveillance, tumors can prevent the receptor recognition by proteolytic cleavage of MICA/B from the cell surface, generating soluble MICA/B in a process called MICA/B shedding. In addition, soluble MICA/B can bind to NKG2D, which in turn induces NKG2D internalization and degradation [128].

Avoiding MICA/B shedding has been investigated as a potential target for cancer immunotherapy. Ferrari de Andrade et al. inhibited MICA/B shedding by blocking the initiation of release through antibodies binding to key epitopes on the MICA and MICB α3 domain (a membrane-proximal domain). Such binding prevents the shed action of the proteases but does not interfere with the interaction of NKG2D with the α1 and α2 domains of MICA (Fig. 2b) [129]. The study reported that after treatment with MICA α3 domain-specific antibodies there was an increase in the binding of NKG2D to target ligands, thus inducing greater tumor immunity mediated by NK cells. This strategy could be applied in combination with CAR-NK-cells to additionally support their activity.

### NK-cells CD38KO

Recently, the high expression of CD38 on malignant cells has prompted the development of targeted immunotherapies, especially in multiple myeloma (MM) [130]. The FDA has approved a new immunotherapy for MM, a mAb targeting CD38, called daratumumab (DARA) [131]. Despite the well-established clinical benefits of DARA, some patients experience disease relapse, and a possible explanation is the rapid depletion of NK cells after treatment with DARA since NK cells also express relatively high levels of CD38 [132,133]. The reduction of circulating CD38^+^ NK cells results in an inefficient ADCC against MM cells [134].

Aiming to overcome this problem, Kararoudi et al. proposed to delete CD38 in NK cells, by using a DNA-free method with Cas9 ribonucleoprotein complexes (Cas9/RNP) and associated this CD38 knockout (KO) NK cells with DARA in the treatment (Figure 2c) [135]. The authors reported that these cells were resistant to DARA-induced conjugation and fratricide, and persisted in the presence of DARA *in vivo*, in addition to showing superior ADCC activity against MM cell lines and primary samples when compared with the paired CD38 wild-type cells.

### NK-cells engineered to express IL-15 receptor fusion

Recently, strategies for cancer treatment using IL-15 alone or associated with other therapies have been reported [136, 137]. It was demonstrated that soluble IL-15 treatment can induce NK and CD8 T-cell proliferation in patients, but toxicities were reported [136,138]. Studies demonstrated that the binding of IL-15 to the IL-15 alpha subunit (IL-15Rα) is important to its function and prolongs its half-life in circulation [139, 140]. In addition, animal models and Phase 1 clinical trials have shown that IL-15/IL-15Rα complex is less toxic than IL-15 and was not associated with severe adverse events [137, 141, 142].

IL-15 secreting CAR19-NK-cells were successfully developed by Rezvani’s group. In this study, 73% of patients with CD19^+^ tumors responded to CAR19-NK cells treatment and serious adverse effects were not reported [125]. Recently, Ma et al. demonstrated that the combination of oncolytic virus expressing IL-15/IL-15Rα sushi domain fusion protein with EGFR-CAR NK-cells improved efficiency and prolonged survival compared to EGFR-CAR NK cells alone in a glioblastoma model. Interestingly, soluble IL-15/IL15-Rα also improves CAR-NK persistence without inducing exhaustion [143].

According to these results, an interesting strategy for cancer treatment would be the development of NK-cells expressing membrane-bound IL-15/IL-15Rα superagonists (Fig. 2d). Indeed, CAR-NK for different targets (such as CD19, MICA/B, B7H3, and BCMA) in combination with IL-15/IL-15Rα have been tested in pre-clinical studies [137,144].

Valamehr’s group has successfully worked on multi-engineered CAR-NK-cells for MM treatment. These iPSC-NK cells are specific for BCMA and besides a membrane-bound IL-15/IL-15Rα complex, they are KO for CD38 and express ncCD16A [145,146]. An anti-CD19 CAR engineered with IL-15/IL-15Rα and ncCD16A has also been developed with iPSC-NK cells, a clinical trial for dose determination of this construct to be used alone or in combination with anti-CD20 mAb is currently recruiting patients (NCT04245722) (Table 2) [147].

### NK CISH KO

The edition of the *CISH* gene is another way of exploring the role of IL-15 in NK activation and mitigating the toxicities associated with intravenous IL-15 application (Fig. 2e). CIS is an inhibitory intracellular protein that blocks the binding of STATs to cytokine receptors [148, 149]. Therefore, due to its role as an immune checkpoint inhibitor, recent studies have explored the potential of knocking out the *CISH* gene to enhance NK cell sensitivity to IL-15.

Bernard and collaborators developed a conditional mouse model for *CISH* gene depletion in NK cells to better understand its role in NK regulation. *CISH* depletion did not affect the maturation or immunophenotypic profile of NK cells, but NK-cells over-express genes associated with cell-cycling and activation, resulting in increased production of IFN-γ and CD107a expression. Interestingly, *CISH* KO NK cells were able to proliferate at a low concentration of IL-15 *in vitro*, demonstrating more sensitivity to IL-15. The conditional depletion of *CISH* in NK cells improves *in vivo* response against breast cancer cells and decreases TIGIT expression, a receptor that is associated with NK cell exhaustion [149]. Similar results were achieved in human *CISH* KO iPSC-NK cells by Kaufman’s group. *CISH* KO iPSC-NK cells expanded at low IL-15 levels *in vitro* and presented better antitumor response and long persistence *in vivo* [150].

Recently, Daher and collaborators demonstrated that *CISH* KO CAR19-NK-cells secreting IL**-**15 had enhanced antitumor response compared to IL-15-secreting CAR19-NK *in vitro* and *in vivo*. Depletion of the *CISH* gene resulted in increased expression of activator receptors and proteins related to cytotoxicities, such as granzyme B, perforin, TRAIL, CD3z, DAP12, DNAM**-1**, CD25, and Ki67. The RNA-sequencing analysis also demonstrated the upregulation of genes of tumor necrosis factor TNF and IFN signaling and genes of cytokine signaling after *CISH* KO. It is important to note that treatment with *CISH* KO CAR19-NK did not result in toxicities or abnormal NK expansion and that it was dependent on the IL-15 gene in the CAR construct, suggesting it is a safe product for clinical use [148].

### NK-cells engineered with modified TGF-β receptor

Targeting the TGF-β receptor is another approach to enhance the cellular metabolism and the antitumor response of NK cells. TGF-β is an important cytokine for cell differentiation, migration, apoptosis, wound healing, and angiogenesis. It has been postulated that TGF-β suppresses NK function by reprogramming the metabolism [151]. Thus, different strategies for modulation of TGF-β signaling in NK cells have been proposed.

Recently, Yvon et al. developed NK-cells engineered to express a TGF-β-dominant-negative receptor II (DNRII), which resulted in the inhibition of TGF-β signaling. *In vitro* studies demonstrated that DNRII CB-NK-cells can efficiently kill tumor cells and can increase the expression of perforin, IFN-γ, NKG2D, and DNAM-1, even in the presence of TGF-β [152]. Animals with lung metastasis treated with NK-cells expressing DNRII had similar results showing decreased tumor growth [153].

Furthermore, the construction of a chimeric TGF-β receptor coupled to activating molecules has also been shown to be a promising strategy (Fig. 2f). NK-cells engineered to express TGF-β type II receptor coupled to the NKG2D intracellular domain, or a receptor containing the truncated TGFβRII domain linked to the synthetic Notch-like receptor coupled to the transcription factor RELA (which activates NK cells), had their antitumor activity enhanced [154, 155].

## Conclusion

Even though there are many difficulties in developing CAR-NK-cells, a lot of improvements in their manufacturing have been made. We are at the moment testing several methods to expand and activate primary NK-cells, among these the use of feeders, beads, and cytokines enables the production of enough cells for clinical use. In addition to viral vectors, non-viral delivery methods are being optimized to produce a safer option for clinical use, including electroporation and/or cell squeezing. Non-viral vectors such as transposons, episomes, and CRISPR-Cas9, have been successfully implemented in pre-clinical studies and mRNA is already undergoing CAR-NK clinical trials.

Regarding CAR structure, the use of NK-specific signaling molecules in CAR signaling domains is a strategy that enables us to increase the cytotoxicity of CAR-NK cells. Furthermore, we have presented several engineering strategies that might help to increase CAR-NK-cell antitumor efficiency. Stabilizing MICA/B in the tumor cell membrane in combination with CAR-NK-cells has not yet been tested, but this could be an interesting approach for treating MICA/B^+^ tumors. The use of chimeric receptors to convert TGF-β inhibitory signaling into an activating signal could help to escape microenvironment TGF-β inhibition. CAR-NK-cells combining the CD38 KO with ncCD16A and also expressing membrane-bound IL-15/IL-15Rα are already being tested. The expression of IL-15/IL-15Rα is an interesting approach due to its capacity to increase proliferation, efficiency, and persistence of CAR-NK cells, which can be further improved by *CISH* KO.

Considering the many advances in CAR-NK-cells engineering, their clinical application as an off-the-shelf product is imminent. We are heading to a future where non-viral vectors may become the main delivery method for CAR expression, and NK cells can permit a ready-to-use targeted cell therapy with a safer profile. Taking advantage of the several existing engineering options can allow to develop more robust CAR-NK-cells. Moreover, their combination with other therapies such as monoclonal antibodies against CTLA-4 or PD-1/PDL-1 could increase CAR-NK-cells effector function. Therefore, CAR-NK-cells could allow more cancer patients to benefit from cell therapy.

## Data Availability

Statement: No data available as this is a review article.

## Abbreviations

AAV: Adeno-associated virus
ADCC: Antibody-dependent cell-mediated cytotoxicity
AML: Acute myeloid leukemia
BaEV-LV: Baboon envelope pseudotyped lentiviral vectors
CAR: Chimeric antigen receptor
CB: Cord blood
CLL: Chronic lymphocytic leukemia
CRS: Cytokine release syndrome
GvHD: Graft versus host disease
MP: Good manufacturing practice
HLA: Human leucocyte antigen
IL: Interleukin
iPSC: induced pluripotent stem cells
ITAM: Immunoreceptor tyrosine-based activation motif
ITR: inverted terminal repeat sequences
KO: Knockout
KIR: Killer-cell immunoglobulin-like receptor
LDLR: Low-density lipoprotein receptors
mAb: monoclonal antibody
MM: Multiple myeloma
NK: Natural killer
PB: Peripheral blood
PBMC: Peripheral blood mononuclear cell
RV: Retroviral vector
SB: Sleeping beauty
scFv: single-chain variable fragment
S/MAR: Scaffold/matrix attachment region
TM: Transmembrane domain
TME: Tumor microenvironment
VSV-G: Vesicular stomatitis virus type-G
